# A novel lncRNA PTTG3P/miR-132/212-3p/FoxM1 feedback loop facilitates tumorigenesis and metastasis of pancreatic cancer

**DOI:** 10.1038/s41420-020-00360-5

**Published:** 2020-11-30

**Authors:** Wenyu Liu, Jian Tang, Huiqing Zhang, Fanyang Kong, Huiyun Zhu, Ping Li, Zhaoshen Li, Xiangyu Kong, Kaixuan Wang

**Affiliations:** 1 Department of General Surgery, Changhai Hospital, Second Military Medical University, Shanghai, 200433 China; 2 Department of Gastroenterology, Changhai Hospital, Second Military Medical University, Shanghai, 200433 China; 3 Department of Gastrointestinal Medical Oncology, Jiangxi Cancer Hospital, Nanchang, Jiangxi 330029 China

**Keywords:** Cancer, Oncogenes

## Abstract

Pseudogene pituitary tumor-transforming 3 (PTTG3P) is emerging as a key player in the development and progression of cancer. However, the biological role and clinical significance of PTTG3P in pancreatic ductal adenocarcinoma (PDAC) remain unclear. Here, we found that PTTG3P was significantly upregulated in PDAC tissues. Elevated PTTG3P expression correlated with larger tumor size and worse differentiation, and reduced overall survival. Bioinformatics and experimental evidence revealed that PTTG3P promoted malignant phenotypes and FoxM1 signaling pathway in PDAC cells. Mechanistically, PTTG3P functions as a microRNA sponge to positively regulate the expression of FoxM1 through sponging miR-132/212-3p. Moreover, it showed that FoxM1 transcriptionally activated PTTG3P expression, thus forming a feedback loop to promote the aggressiveness of PDAC cells. Taken together, our findings suggest that PTTG3P promotes PDAC progression through PTTG3P/miR-132/212-3p/FoxM1 feedforward circuitry and it may serve as a promising diagnostic marker or target for treatment in PDAC patients.

## Introduction

Pancreatic cancer is one of the most lethal diseases of all human malignancies worldwide and ranks sixth in cancer-related deaths. It has a 5-year survival rate of less than 6% and a median survival rate of about 6 months. Pancreatic ductal adenocarcinoma (PDAC) is the most common type that accounts for more than 90% of cases^1^. A combination of factors leads to poor prognosis of PDAC, including difficulties in detecting early stage disease, high locally advanced or distant metastatic potential, and resistance to conventional therapies. Thus, better understanding of the initial events in PDAC development and the development of innovative targeted molecular therapies for this disease is urgently needed.

Pseudogenes, as a type of long noncoding RNA (lncRNA), have attracted great research interest. Human genome is estimated to contain more than 18,000 pseudogenes. Pseudogenes have emerged as a previously unappreciated class of sophisticated modulators of gene expression, with a multifaceted involvement in the pathogenesis of human cancer. Recently, mounting evidence demonstrated that dysregulated expression of pseudogenes and alterations of their interactions with other biological molecules could regulate a diverse set of human cancer pathological processes, including proliferation, metastasis, metabolism, apoptosis, drug-resistance, and cancer-related inflammation^2–4^. Examples are CYP4Z2P^5^, NANOG^6^ and PTENP1^7^. Given the pivotal and multifaceted role that pseudogene played in human cancer, it offers strong rationale for further systematic study to uncover the key pseudogene regulators and the involved crucial pathway signaling during carcinogenesis.

Pituitary tumor-transforming 3, pseudogene (PTTG3P), is a processed pseudogene that shows high homology to its parental gene, PTTG1 and PTTG2^8^. Pituitary tumor-transforming genes, especially PTTG1, has long been recognized as a marker of invasive cancer and potential therapeutic target^9,10^. Recently, PTTG3P has been demonstrated to be frequently upregulated in various cancers and contribute to clinical aggressiveness^8^. Mechanical investigation revealed PTTG3P could facilitate tumor growth and metastasis by interacting with microRNAs or proteins^8,11,12^. However, its expression, biofunction and clinical significance in PDAC are yet to be determined.

Here, we present for the first time a detailed analysis of PTTG3P’s clinical significance and functional role in PDAC progression. It showed that PTTG3P is highly expressed in PDAC tissues and predicts poor prognosis. Elevated PTTG3P was associated with larger tumor size and poorer differentiation. Functional study revealed PTTG3P promote tumor growth and metastasis *via* functioning as a ceRNA for miR-132/212-3p, thereby preventing its association with target FoxM1 mRNA. Additionally, we found PTTG3P could be transcriptionally activated by FoxM1, thus forming a potent feedforward circuitry to enhance PTTG3P-induced pro-tumor effect. These results indicated that PTTG3P exert oncogenic potential and may be a promising candidate for prognosis and therapy in PDAC patients.

## Material and method

### Patients and tissue specimen

Two independent cohorts involving 85 PDAC patients were enrolled in this study. In cohort 1, tissue microarray (TMA) containing 25 PDAC specimens from Changhai hospital was constructed. In cohort 2, 60 paired fresh tumor tissues and adjacent normal tissues were obtained from patients who had undergone routine surgery in Changhai hospital. All patients had been definitely diagnosed with PDAC by surgery and pathological examination. None of these patients had received radiotherapy or chemotherapy before surgery. Tissue samples were immediately frozen in liquid nitrogen as soon as it was collected from patients and stored at −80 °C. The human materials were obtained with consent of patients and approved by Ethics Committees of Changhai Hospital. Patients’ clinical information of these two cohorts were listed in Supplementary Tables 1 and 2.

### In situ hybridization (ISH) and immunohistochemical analysis

ISH was performed using the ISH Kit (Boster Bio-Engineering Company, Wuhan, China) according to the manufacturer’s instructions. Immunohistochemical analyses of these specimens were conducted with antibody against FoxM1 (cat. # Ab137647, Abcam) as described previously^13^. The ISH staining for PTTG3P and IHC staining for FoxM1 were scored by 2 pathologists blinded to the clinical parameters. The final score for each ISH and IHC staining was calculated with the graded staining percentage and intensity, ranging from 0 to 12. This scoring system is relatively reproducible and gives highly concordant evaluations between independent pathologists, as described in our previous studies^14^.

### Cell lines and cell culture

The HPNE, 293 T and all the human pancreatic cancer cell lines (AsPC-1, BxPC-3, CaPAN-2, MiaPaCa-2, PANC-1, SW1990) were obtained from American Type Culture Collection. All of these cell lines were maintained in plastic flasks as adherent monolayers in Eagle’s minimal essential medium supplemented with 10% fetal bovine serum, sodium pyruvate, nonessential amino acids, L-glutamine, and a vitamin solution (Flow Laboratories). All cells were incubated at 37 °C in a humidified atmosphere with 5% CO_2_.

### Oligonucleotides and transfection

PTTG3P short hairpin RNA (shRNA) and FoxM1 small interfering RNA (siRNA) were chemically synthesized by GenePharma (Shanghai, China). The shRNA and its corresponding control sequences were inserted into the lentivirus vector pLVTH (GenePharma, Shanghai, China). MiR-132/212-3p mimics, miR-132/212-3p inhibitor and related negative controls (NC) were bought from GenePharma (Shanghai, China). The oligonucleotides were transfected into PDAC cells using Lipofectamine 2000 (Invitrogen, USA) and the transfection efficiency was confirmed by qRT-PCR.

### Quantitative real-time PCR (qRT-PCR)

Total RNA was isolated with using Trizol reagent (Invirogen, MA, USA). Quantitative PCR was performed using SYBR-green mastermix (TaKaRa, Shiga, Japan) based on manufacturer’s instructions and run in a LightCycler 480II instrument (Roche, Mannheim, Germany). GAPDH mRNA was employed as an endogenous control for mRNA and lncRNA. To detect the miR-132/212-3p level, the special primer was obtained from RiboBio (Guangzhou, China), and U6 was used for normalization. PCR primers are listed in Supplementary Table 3.

### Western blot analysis

Western blotting was performed using a SDS-PAGE Electrophoresis System according to the previous description with primary antibodies specific for FoxM1 (1:2000, Abcam, cat. # ab137647, UK), PTTG1 (1:2000, Abcam, cat. #ab79546, UK) and Dicer (1:2000, Cell Signaling T, cat. #5362 S); and a secondary antibody (anti-rabbit IgG or anti-mouse IgG; Santa Cruz Biotechnology, USA). Equal protein sample loading was monitored using an anti-GAPDH (1:2500, Abcam, cat. # ab181602, UK) antibody.

### Cell counting kit-8 (CCK-8) assay

Cells were seeded in 96-well plates at the density of 2 × 10^3^ cells/well. After 24 h, 10 μl Cell Counting Kit-8 (CCK-8, Dojindo, Japan) was added to each well and cells were then incubated at 37 °C for 2 h in an incubator. After the incubation, phosphate-buffered saline (PBS) was applied to wash the plates twice. The absorbance was then read at 450 nm using a microplate reader according to the manufacturer’s instructions.

### Cell migration/invasion assay

For the Boyden chamber assay, 24-well tissue culture plates with 12 cell culture inserts (Millipore) were used. Each insert contained an 8-μm-pore-size polycarbonate membrane with a pre-coated thin layer of a basement membrane matrix (ECMatrix for the invasion assay) or without a coated matrix (for the migration assay). Ten percent fetal bovine serum-containing medium was placed in the lower chambers to act as a chemoattractant. Cells (5 × 10^5^) in a 300-μL volume of serum-free medium were placed in the upper chambers and incubated at 37 °C for 48 h. Cells on the lower surface of the polycarbonate membrane, which had invaded the ECMatrix and migrated through the membrane, were stained, counted, and photographed under a microscope.

### Scratch wound healing assays

PDAC cells were seeded into the 6-well plates and cultured until they fully fused. The cell monolayer was then manually scratched by a 20-μl pipette tip and washed out with phosphate-buffered saline (PBS). After that, cells were cultured in culture medium supplemented with 1% FBS for at least 12 h. The phase contrast microscope was used to capture the images and the Image Pro Plus v6.0 software package (Media Cybernetics Inc., Bethesda, MD, USA) was used to measure the migration areas of cells.

### In vivo assay

Five-week-old female BALB/C athymic nude mice were kept under specific pathogen-free conditions. The animal experiment was approved by the Animal Research Ethics Committee of Second Military Medical University. To measure tumor growth, PDAC cells (1 × 10^6^) in 0.1 ml of Hank’s balanced salt solution were injected subcutaneously on the left thigh of nude mice. Tumor volume were examined every week and then measured as length × width^2^ × 0.5. All the mice were killed and their tumors were removed 4 weeks after injection. For metastasis evaluation, PDAC cells (1 × 10^6^) were injected intravenously into another group of mice *via* the ileocolic vein. All mice were sacrificed on day 28 after injection or when they appeared to be moribund. Their livers were then removed, and surface metastases on the livers were counted.

### Bioinformatics analysis

The Ualcan (http://ualcan.path.uab.edu/) and LinkedOmics database (http://www.linkedomics.org/) were used as web-based platform for analyzing TCGA cancer-associated multi-dimensional datasets. Both websites allowed a flexible exploration of associations between molecular or clinical attributes of interest. A GEO database (Accession Number GSE65821) was used to analyze gene expression in gastric cancer. The shared microRNAs between PTTG3P and FoxM1 was predicted by miRcode (http://www.mircode.org/index.php) and Targetscan (http://www.targetscan.org/vert_72/). Putative binding sequences of FOXM1 in PTTG3P promoter were obtained from The Animal Transcription Factor DataBase (AnimalTFDB) (http://bioinfo.life.hust.edu.cn/AnimalTFDB/#!/).

### RNA immunoprecipitation (RIP)

RIP was implemented by a Magna RIP™ RNA-Binding Protein Immunoprecipitation Kit (Millipore, Germany). PDAC cells with indicated transfection were harvested and then lysed in the lysis buffer (50 mM Tris-HCl, pH = 7.4, 150 mM NaCl, 1% Triton-100, 0.1% SDS, 1.5 mM EDTA). Thereafter, PDAC cells were lysed and incubated with protein A magnetic beads. The beads were conjugated with Ago2 antibody (Abcam, Cambridge, UK) or anti-IgG (Abcam) as negative control at 4°C for 6 h. Then, the samples were digested applying Dnase I and Proteinase K, followed by the isolation of immunoprecipitated RNA. Finally, immunoprecipitated RNA was subjected to qRT-PCR analysis to demonstrate the presence of PTTG3P.

### MS2-RIP

Full-length PTTG3P (PTTG3P-WT) or mutant PTTG3P (PTTG3P-Mut, mutant in miR-132/212-3p binding site) was cloned into the eukaryotic expression vector pSL-MS2-12X. And then these two plasmids were separately transfected into PDAC cells with MS2-GFP plasmid. After 48 h, RIP assay was performed to detect the direct interaction between PTTG3P and the miR-132/212-3p. IgG and GFP antibodies (Abcam, UK) were used along with the Magna RIP RNA-Binding Protein Immunoprecipitation Kit (MilliporeSigma, Germany).

### Dual luciferase assay

The complementary DNA fragment containing the wild-type or mutant PTTG3P fragment and 3′-untranslated region (UTR) of FoxM1 was subcloned downstream of luciferase gene within the pGL3-Basic luciferase reporter vector (Promega, USA). Sequence analysis of the PTTG3P promoter uncovered four putative FBSs located at −1009 (FBS1), −1001 (FBS2), −993(FBS3) and −192 (FBS4) bp relative to the transcriptional start site of PTTG3P. A full-length PTTG3P promoter PTTG3P-1009 (Covering all FBSs) and a promoter with a deletion mutants (PTTG3P-192, covering FBS4) of it were generated. To determine FoxM1 as upstream regulator of PTTG3P, PDAC cells were transfected with reporters, siRNAs, or specific gene expression plasmids. Relative luciferase activities of 3′-UTR of FoxM1 and PTTG3P were normalized to Renilla luciferase activity at 24 h after transfection. The data are relative to the fold change of pair-matched control groups, which was defined as 1.0.

### Chromatin immunoprecipitation assay

The experiments were performed according to previous reports. PDAC cells (2 × 10^6^) were prepared for a chromatin immunoprecipitation (ChIP) assay using a ChIP assay kit (Millipore) according to the manufacturer’s protocol. The resulting precipitated DNA specimens were analyzed using PCR to amplify fractions of the PTTG3P promoter. The PCR products were resolved electrophoretically on a 2% agarose gel and visualized using ethidium bromide staining.

### Statistical analysis

Data were exhibited as mean ± standard deviation (SD) from triplicated independent experiments. GraphPad Prism software (Intuitive Software for Science, San Diego, CA) and SPSS 17.0 software (SPSS Inc., Chicago, IL, USA) were used for statistical analyses. The difference of two independent groups was analyzed by a two-tailed Student’s t test, while multigroup comparison was made by ANOVA. Expression correlation between genes was analyzed by Pearson correlation analysis. Survival analysis was conducted using the Kaplan–Meier method and analyzed by the log-rank test. In all of the tests, *P* values less than 0.05 were considered statistically significant.

## Results

### PTTG3P is overexpressed in PDAC and indicates poor prognosis

**Fig. 1:**
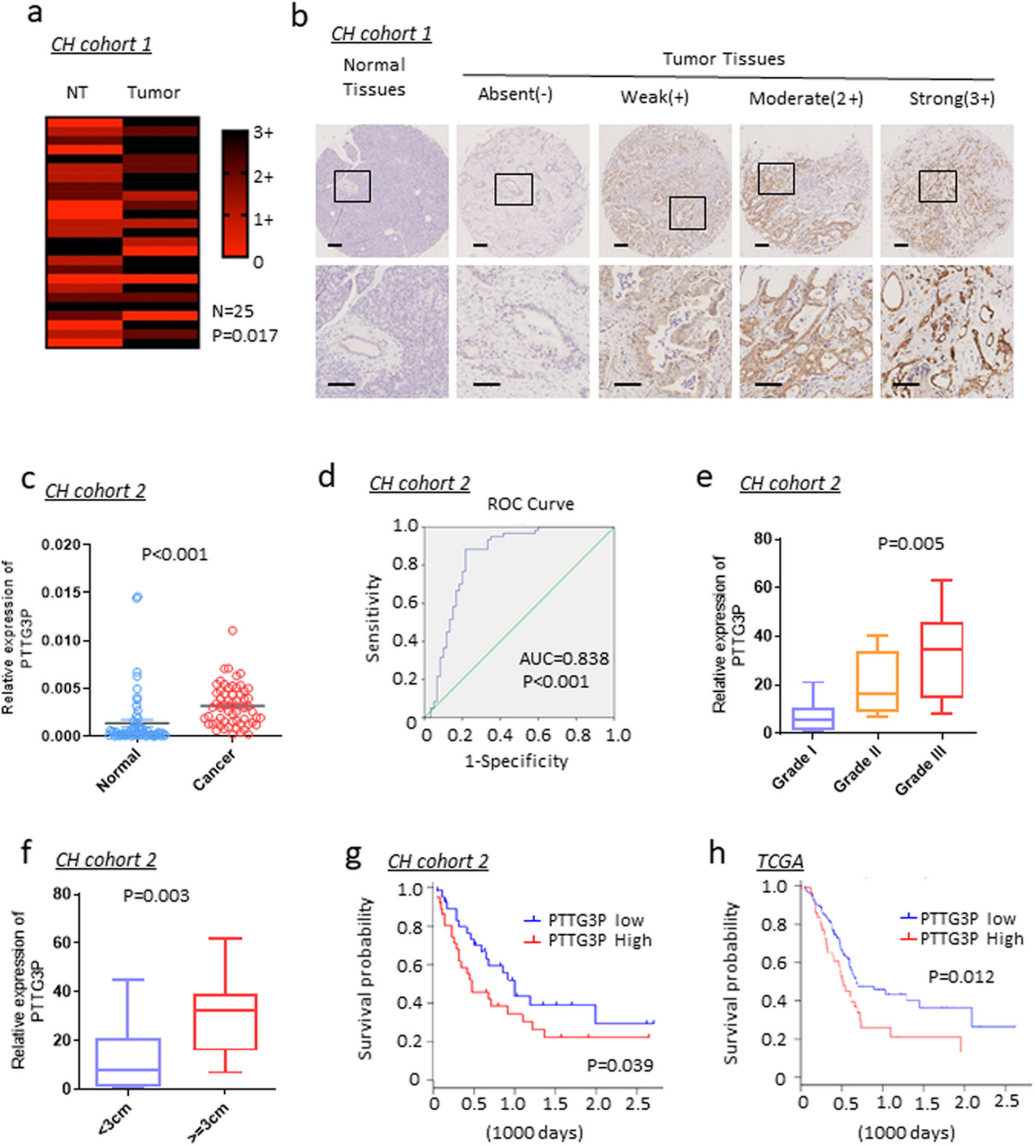
PTTG3P is upregulated in PDAC tissues. **a** In situ hybridization (ISH) analysis of PTTG3P was performed in 25 paired paraffin-embedded PDAC and adjacent tissues (CH cohort 1). Differential expression of PTTG3P in tumor and adjacent tissues was shown in a heat-map and was statistically analyzed by Wilcoxon signed rank tests. **b** Representative images are shown for absent, weak, moderate and strong expression of PTTG3P. Scale bars, 100 μm. **c** PTTG3P expression was analyzed by qRT-PCR in PDAC samples and adjacent non-tumor PDAC tissues from cohort 2 (*N* = 60). PTTG3P expression level was normalized to that of GAPDH. **d** ROC curve for prediction of PDAC using qRT-PCR-based PTTG3P expression level. The AUC was 0.838, with indicated *P* value. e and f. Expression of PTTG3P was positively correlated with grades of tumor differentiation (**e**) and tumor size (**f**). **g** Kaplan–Meier survival analysis of overall survival in PDAC patients in CH cohort 2. **h** UALCAN database showed high expression of PTTG3P indicates poor prognosis of PDAC patients in TCGA cohorts.

Firstly, we detected PTTG3P expression by in situ hybridization (ISH) in a 25 paraffin-embedded PDAC and adjacent tissues (cohort 1, Supplementary Table 1). PTTG3P expression was significantly higher in PDAC than adjacent tissues (Fig. 1a, b, *P* = 0.017). To further validate the correlation of PTTG3P expression in PDAC with clinical characteristics, we examined expression of PTTG3P in 60 paired PDAC tissues and adjacent normal tissues by qRT-PCR (cohort 2). It demonstrated that expression of PTTG3P in PDAC tissues was obviously elevated relative to that in normal controls (Fig. 1c) (*P* < 0.001). Next, paratumorous tissues was used as a control to produce a receiver operating characteristic curve. The analytic result showed that area under the receiver operating characteristic curve was 0.838 (*P* < 0.001) (Fig. 1d). Furthermore, PTTG3P was found to be closely related with differentiation grades (Fig. 1e, *P* = 0.005) and tumor size (Fig. 1f, *P* = 0.003) (Supplementary Table 2). Kaplan–Meier analysis demonstrated that higher expression of PTTG3P correlated with reduced overall survival (OS) (*P* = 0.039) in PDAC patients (Fig. 1g). Similar results was obtained by analyzing TCGA database UALCAN (*P* = 0.012. Fig. 1g). Collectively, these findings suggested the involvement of PTTG3P in cancer development and indicated that PTTG3P may play an important role as a clinical biomarker for PDAC patients.

### Inhibition of PTTG3P attenuates proliferation, migration and invasion of PDAC cells in vitro

**Fig. 2:**
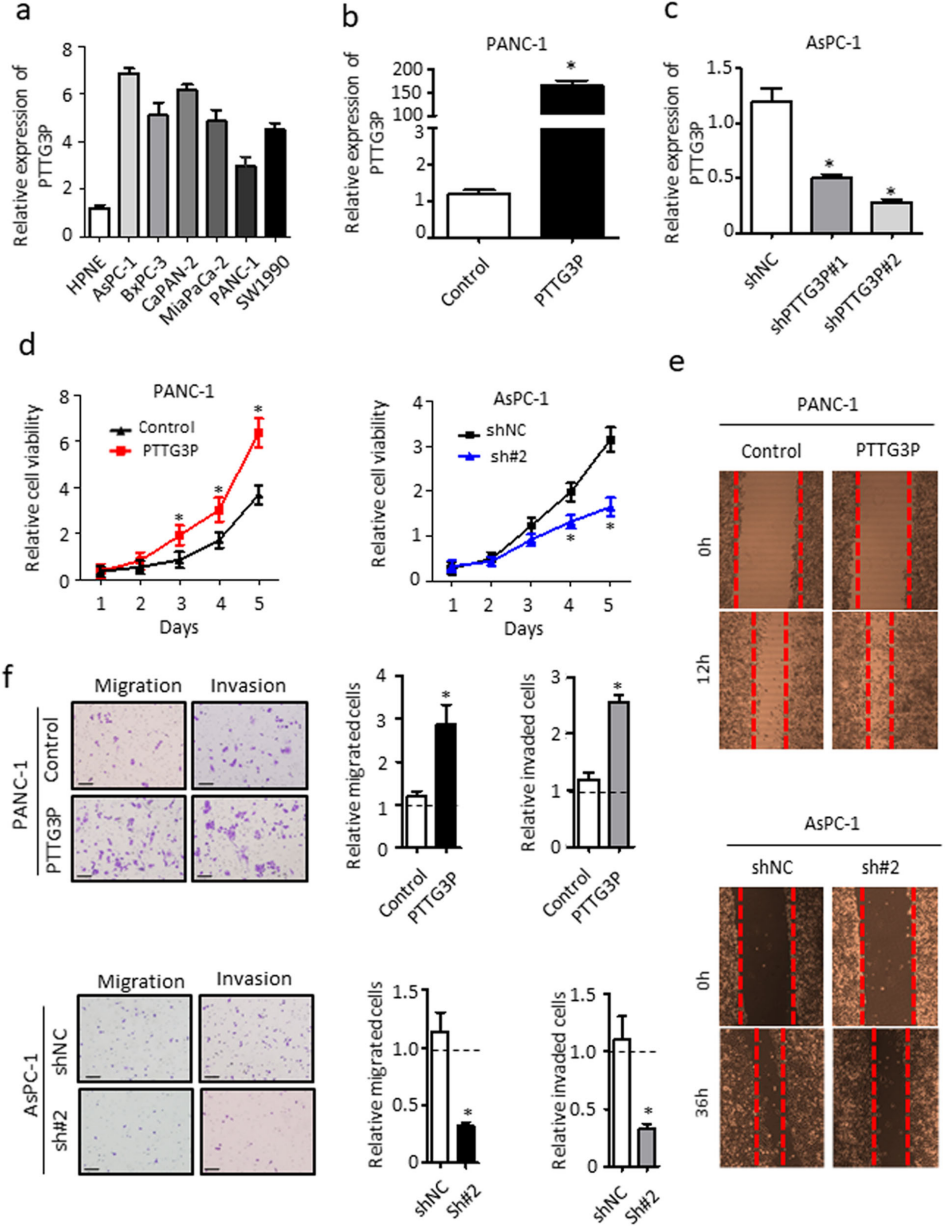
PTTG3P promotes proliferation, migration and invasion of PDAC cells in vitro. **a** PTTG3P expression level were detected in normal pancreatic cell (HPDE) and PDAC cell lines (AsPC-1, BxPC-3, CaPAN-2, MiaPaCa-2, PANC-1 and SW1990). **b** PTTG3P expression examined by qRT-PCR in PANC-1 cells transfected with PTTG3P overexpression plasmid. **c** PTTG3P expression examined by qRT-PCR in AsPC-1 cells transfected with shRNA specific for PTTG3P (#1 and #2). **d** Assessment of PDAC cell growth in vitro by cell counting kit-8 (CCK-8) at the indicated time points. **f** Transwell assays were used to determine the effects of PTTG3P on the migration and invasion ability of PDAC cells. Scale bars, 50 μm. **e** Enforced PTTG3P impaired the migration of tumor cells, whereas knockdown of PTTG3P did the opposite in wound healing assays. *P < 0.05, Means ± SD was shown. The experiments were performed in triplicate.

We evaluated PTTG3P in 7 pancreatic cell lines and found PTTG3P was significantly upregulated in PDAC cell lines than that in nonmalignant cell line (HPNE) (Fig. 2a). The elevated expression pattern suggested that PTTG3P might be involved in driving PDAC progression. To examine the oncogenic functions of PTTG3P in PDAC cells, we established PTTG3P-overexpressed PANC-1 and PTTG3P-knockdown AsPC-1 cell lines respectively. Transfection efficacy was validated by qRT-PCR (Fig. 2b, c). For a better knockdown efficacy, we chose shPTTG3P#2 for the following biofunction assays. Cell-counting kit-8 assays indicated that exogenous expression of PTTG3P resulted in a significant increase in the proliferation index of PANC-1 cells, whereas knockdown of PTTG3P expression did the opposite in AsPC-1 cells (Fig. 2d). Scratch wound assays and transwell assays further confirmed that both the migratory ability and invasiveness of PTTG3P-transfected PANC-1 cells were much greater than those of corresponding control cells. Conversely, the migratory ability and invasiveness of shPTTG3P-transfected AsPC-1 cells were markedly attenuated relative to the control cells (Fig. 2e, f). Taken together, these data demonstrated that PTTG3P is required for PDAC cell proliferation, migration and invasion, supporting that PTTG3P may function as an oncogene in PDAC cases.

### Inhibition of PTTG3P attenuates tumor growth and metastasis of PDAC cells in vivo

**Fig. 3:**
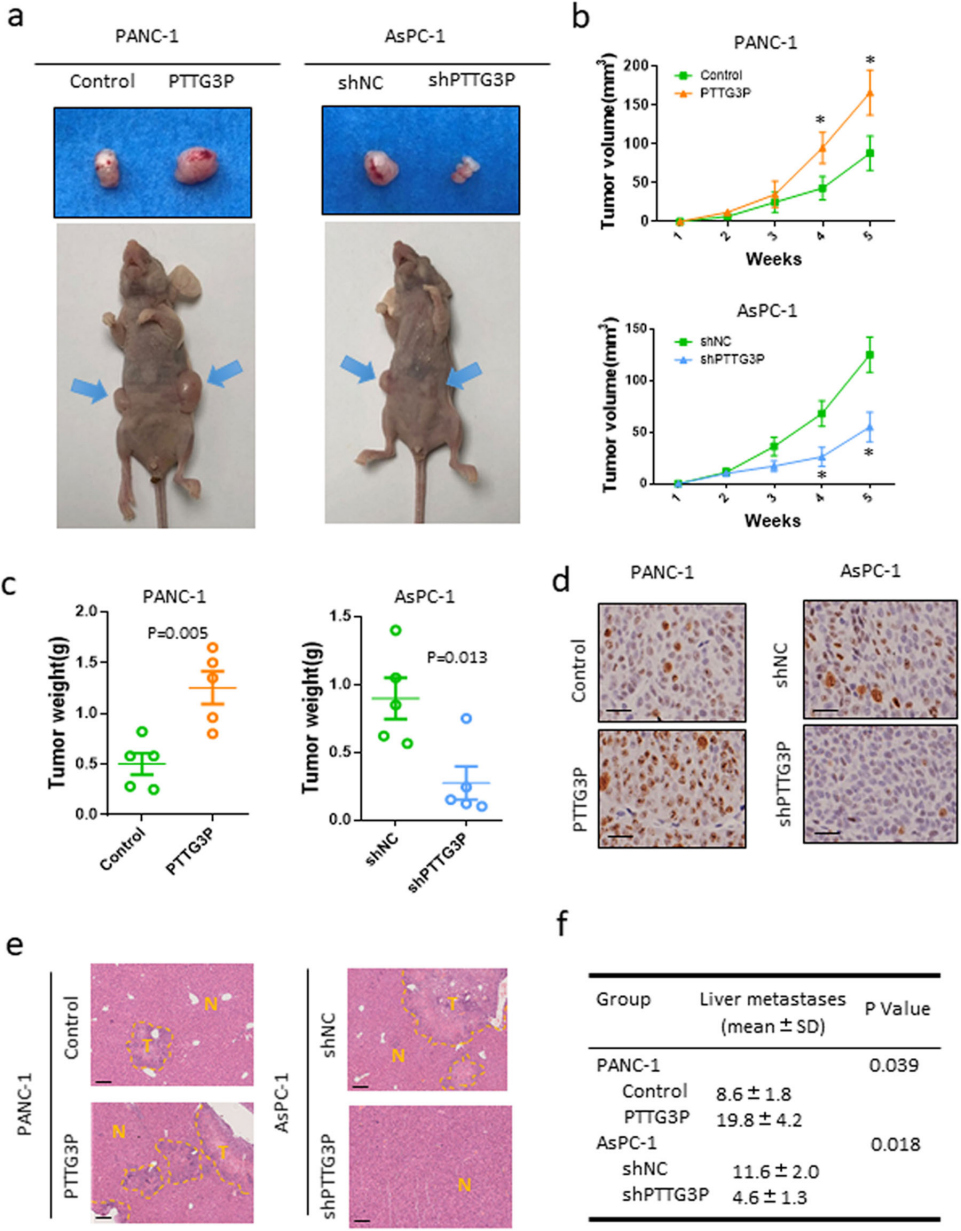
PTTG3P promotes tumor growth and metastasis of PDAC cells in vivo. PTTG3P-overexpressing plasmid or shRNA treated PANC-1 or AsPC-1 cells were injected subcutaneously in the anterior armpit of nude mice (1 × 10^6^ cells per mouse, five mice per group). **a** Gross tumor (a), tumor volume (**b**) and tumor weight (**c**) from indicated groups were shown. The formula to calculate the tumor volume is *ab*^2^/2, *a* represents the longest diameter of the tumor, and *b* represents the diameter perpendicular to the longest. **d** The expression level of Ki67 was examined by IHC analysis. Scale bars, 50 μm. **e** Representative images of HE-stained sections of metastatic nodules in the liver of nude mice (tumor areas are outlined with dashed lines; T tumor). Scale bars, 100 μm. **f** Mean and standard deviation of numbers of liver metastases were calculated. *P < 0.05.

Given that PTTG3P enhances cell proliferation and migration/invasion in vitro, we further assessed the tumorigenic ability of PTTG3P in tumor growth and cancer cell dissemination using xenograft mouse models. For tumor growth model, we subcutaneously transplanted PDAC cells with PTTG3P overexpression or knockdown into 5-week-old nude mice. Tumor burden was monitored once a week. It showed that transfection with PTTG3P consistently promoted the growth of PANC-1 cells in the subcutis, and knockdown of PTTG3P inhibited the growth of AsPC-1 cells (Fig. 3a–c). The tumor cell proliferation ability was examined by IHC assays for Ki-67 protein (Fig. 3d). Tumor metastasis model was established by injecting tumor cells intravenously into mice *via* the ileocolic vein. It revealed that enforced expression of PTTG3P markedly increased hepatic metastasis of PANC-1 cells, whereas downregulation of PTTG3P in AsPC-1 did the opposite (Fig. 3e, f). These data illustrated that PTTG3P promoted the tumor growth and metastasis of PDAC in vivo, further suggesting the essential function of PTTG3P in PDAC development and progression.

### FoxM1 is responsible for PTTG3P-mediated cell proliferation, migration and invasion

**Fig. 4:**
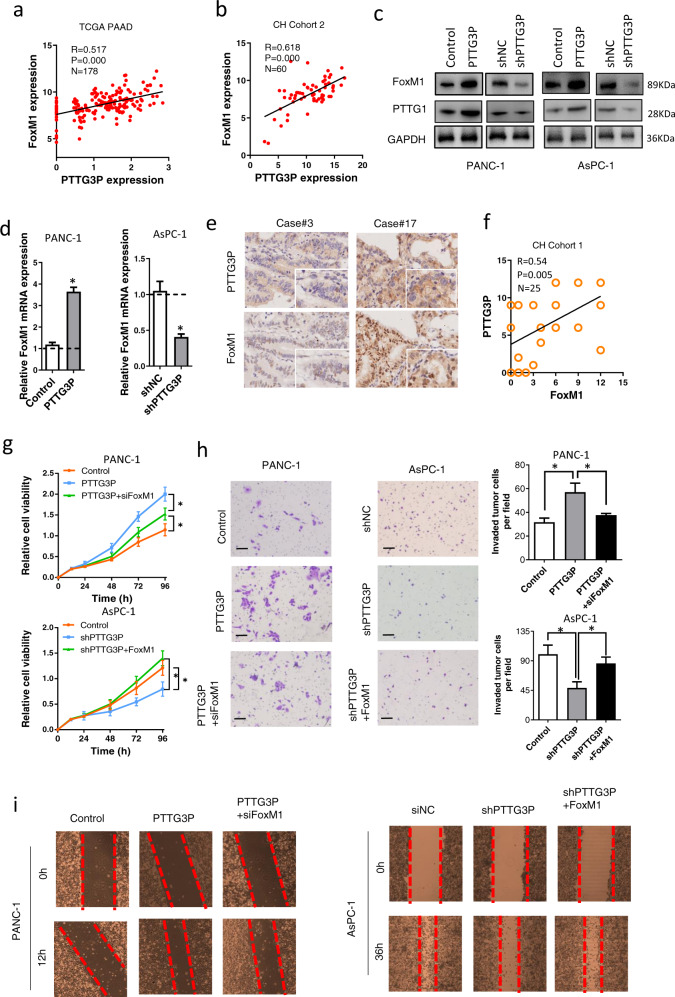
PTTG3P and FoxM1 were concomitantly expressed in PDAC tissues. **a**, **b** PTTG3P transcripts were linearly correlated with FoxM1 mRNA in PDAC tissues from both TCGA (a) and CH cohort 1 (**b**). **c**, **d** Protein (**c**) and mRNA (**d**) expression of FoxM1 in PDAC cells transfected with PTTG3P-overexpressing plasmid or specific shRNA. **e** Analysis of PTTG3P by ISH and FoxM1 by IHC in PDAC tissues from CH cohort 2 (*n* = 25). Representative images of PDAC sections with PTTG3P and FoxM1 staining are shown (×100 magnification in the main images; ×400 magnification in the insets). **f** Assessment of the positive correlation between PTTG3P and FoxM1 expression in PDAC specimens (*n* = 25) using Pearson correlation coefficient analysis. Some of the dots on the graph represent more than one specimen. **g** CCK-8 assay indicated FoxM1 mediated PTTG3P-induced PDAC cell proliferation. **h**, **i** Matrigel invasion (**h**) and wound healing (**i**) assay results for PANC-1 cells after inducing PTTG3P with or without transfection of FoxM1 shRNA and AsPC-1 cells after silencing PTTG3P with or without transfection of FoxM1. Scale bars, 50 μm. *P < 0.05, Means ± SD was shown. The experiments were performed in triplicate.

To gain insight into the molecular mechanism involved in PDAC pathogenesis, we analyzed the expression patterns of PTTG3P and mRNAs according to the results from LinkedOmics database, which has data regarding PDAC in 178 patients incorporated into the TCGA project. In the co-expression network, RNAs were ranked based on Pearson correlation coefficients (PCC) and those ranked from top 50 exhibited comparable correlations (PCC range:0.51~0.63) (Supplementary Fig. 1a, b). Among those identified involved multiple critical regulators for pancreatic carcinogenesis, including FoxM1 (PCC = 0.52, *P* = 0.000) (Fig. 4a). Such correlation was further validated in our cohort 2 PDAC patients (Fig. 4b). Positive correlation between PTTG3P and FoxM1 were also observed in liver cancer (*R* = 0.593, *P* = 0.000), colorectal cancer (*R* = 0.341, *P* = 0.000) and gastric cancer (*R* = 0.900, *P* = 0.000) tissues by analyzing TCGA and GEO databases (Supplementary Fig. 1c). Western blot and qRT-PCR revealed that PTTG3P promoted FoxM1 expression in PDAC cells (Fig. 4c, d). PTTG1, parental gene of PTTG3P, was also observed to be a target of PTTG3P in PDAC cells (Fig. 4c). Furthermore, we analyzed protein level of FoxM1 and transcriptional level of PTTG3P in 25 paired PDAC tissues (cohort 1). It showed FoxM1 was positively correlated with PTTG3P (Fig. 4e, f).

Next, we examined role of FoxM1 in PTTG3P-regulated tumor cell aggressiveness. Modulation efficacy of FoxM1 overexpression plasmid and siRNA were examined by Western blot (Supplementary Fig. 1d). As shown in Fig. 4g, knockdown of FOXM1 in PANC-1 cells could abrogate the effects of PTTG3P on promoting cell proliferation. In parallel, overexpression of FOXM1 significantly rescue the proliferative inhibition of AsPC-1 cells by PTTG3P knockdown. Results from transwell (Fig. 4h) and scratch wound assays (Fig. 4i) confirmed similar role of FoxM1 on PTTG3P-induced pro-tumor biofunctions. These data indicated that PTTG3P-regulated cell aggressiveness at least partially depends on FoxM1 in PDAC.

### PTTG3P serves as a sponge for miR-132/212-3p in PDAC

**Fig. 5:**
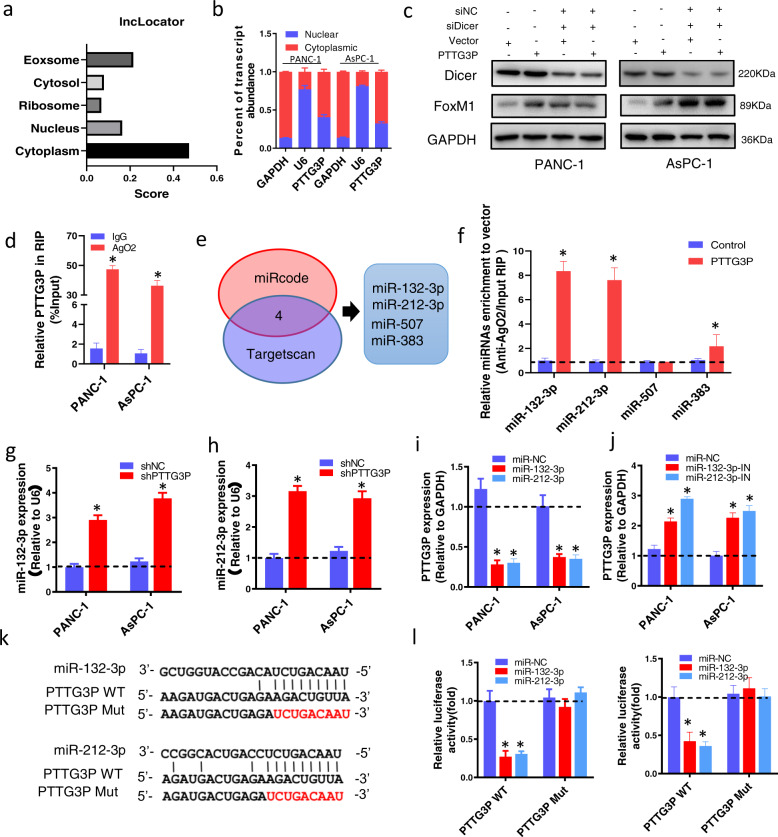
PTTG3P functions as a ceRNA for miR-132/212-3p in PDAC cells. **a**, **b** PTTG3P localization was predicted using lncRNA subcellular localization predictor, lncLocator (**a**) and validated by subcellular fractionation (**b**). **c** Western blot analysis of FoxM1 in PDAC cells transfected with Dicer specific siRNAs and PTTG3P overexpression plasmid. **d** Ago2-RIP assay and RT-PCR were performed in PDAC cells to identify coprecipitated RNA. **e** The Venn diagram identified four microRNAs (miR-132-3p, miR-212-3p, miR-507 and miR-383) in total targeting PTTG3P and FoxM1 3′UTR. The predicted microRNAs from miRcode database (up) targeting PTTG3P transcript were used to overlap those predicted by Targetscan targeting FoxM1 3′UTR. **f** Ago2-RIP assays showed the enrichment of PTTG3P in PANC-1 cells transfected with miR-NC or four predicted microRNAs. **g**, **h** qRT-PCR analysis showed expression change of miR-132-3p (**g**) and miR-212-3p (**h**) with PTTG3P knockdown in PDAC cells. **i**, **j** Expression of PTTG3P in PDAC cells with altered miR-132-3p (**i**) or miR-212-3p (**j**). **k** The predicted miR-132-3p and miR-212-3p binding sites in the PTTG3P transcript. The red nucleotides represent mutant sequences of target sites. **l** Dual-luciferase assays implied that PTTG3P-WT instead of PTTG3P-Mut was targeted by miR-132/212-3p. *P < 0.05, Means ± SD was shown. The experiments were performed in triplicate.

Subcellular location of PTTG3P was predicted by using lncRNA subcellular localization predictor (lncLocator, http://www.csbio.sjtu.edu.cn/bioinf/lncLocator/) (Fig. 5a) software and subcellular fractionation (Fig. 5b), which indicated PTTG3P was mainly localized to the cytoplasm. Certain cytoplasmic lncRNAs are known to serve as microRNAs sponges in the context of cancer, effectively shielding mRNAs from microRNA targeting^15^. To test this hypothesis, we first transfected PDAC cells with Dicer-specific siRNAs to efficiently inhibit microRNA biogenesis. Results showed that PTTG3P-induced upregulation of FoxM1 was abrogated when Dicer was knocked down (Fig. 5c). Immunoprecipitation assay confirmed that PTTG3P bound to Ago2, a main component of microRNA-related silencing complex (Fig. 5d). We then used a bioinformatics analysis to explore the shared microRNAs between PTTG3P and FoxM1 3′-UTR (Fig. 5e, Supplementary Tables 4 and 5). Eventually, the PTTG3P-microRNA-FoxM1 network identified 4 candidate microRNAs in total. Ago-2-RIP assay showed that miR-132-3p and miR-212-3p were the highest enriched microRNAs in PTTG3P overexpression group than the vector group (Fig. 5f). Therefore, we pursued miR-132/212-3p as a primary candidate for further investigation. By analyzing online Ualcan database, we found miR-212-3p, instead of miR-132-3p predicted worse overall survival in PDAC patients (Supplementary Fig. 2). It demonstrated that PTTG3P silencing significantly increased the expression of miR-132/212-3p (Fig. 5g, h). PTTG3P expression was suppressed by overexpression of miR-132/212-3p but promoted by the specific microRNA inhibitors (Fig. 5i, j). Next, we designed reporter construct in which the putative miR-132/212-3p binding sites in the PTTG3P sequence was mutated by site-directed mutagenesis (Fig. 5k). Transfection of miR-132/212-3p mimics significantly inhibited the luciferase activity of reporters containing PTTG3P-WT, instead of PTTG3P-Mut (Fig. 5l). MS2-RIP assay was used to further verify the direct interaction between miR-132/212-3p and PTTG3P. The MS2-tagged wild-type PTTG3P vector was enriched for miR-132/212-3p compared to the empty and mutant plasmids (Supplementary Fig. 3a–c). These data strongly suggested that PTTG3P could sponge miR-132/212-3p in PDAC cells.

### PTTG3P promotes FoxM1 expression by sponging miR-132/212-3p

**Fig. 6:**
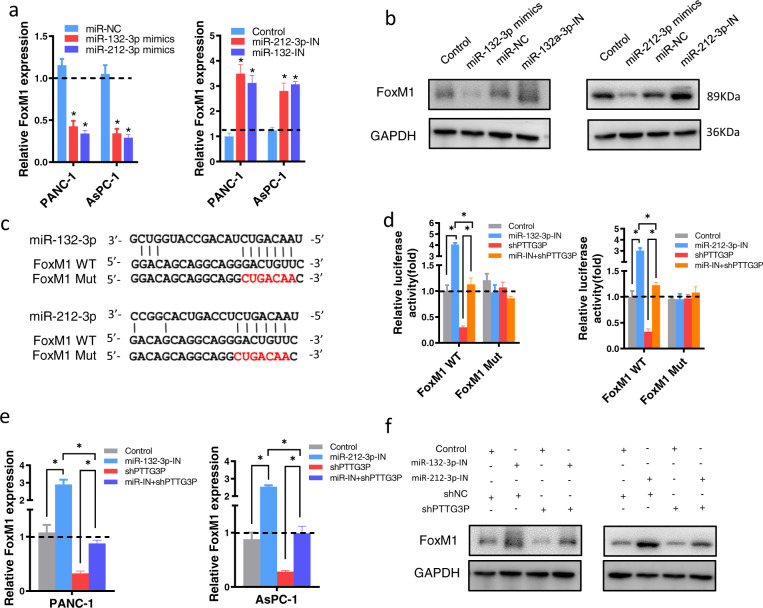
FoxM1 is a target of miR-132/212-3p and indirectly regulated by PTTG3P. **a** MiR-132/212-3p regulated FoxM1 expression in both mRNA (**a**) and protein (**b**) level in PDAC cells. MiR-132/212-3p mimics and inhibitor (IN) were transfected in PDAC cells. The mRNA and protein expression of FoxM1 were examined by qRT-PCR (**a**) and western blot (**b**). c. The predicted binding sites of miR-132/212-3p in the 3′-UTR of FoxM1. The red nucleotides represent mutant sequences of target sites. **d** Dual-luciferase assays implied that miR-132-3p (left) or miR-212-3p (right) inhibitor promoted activity of FoxM1-WT reporter in PANC-1 cells, which could be attenuated by PTTG3P knockdown. Activity of mutant FoxM1 reporter showed no response to alerted miR-132/212-3p or PTTG3P expression. **e**, **f** Transfection of miR-132-3p (left) or miR-212-3p (right) inhibitor promoted FoxM1 expression in mRNA (**e**) and protein (**f**) level, which could be partially attenuated by PTTG3P knockdown. *P < 0.05, Means ± SD was shown. The experiments were performed in triplicate.

Next, we investigated the potential inhibitory effect of miR-132/212-3p on FOXM1 in PDAC cells. As expected, upregulation of miR-132/212-3p reduced the abundance of FoxM1 in PDAC cells at both mRNA and FoxM1 levels, while specific inhibitors for miR-132/212-3p increased FoxM1 in terms of RNA and protein levels (Fig. 6a, b). We constructed luciferase reporters containing wide type and mutated binding sites in 3′-UTR region of FoxM1 (Fig. 6c). It showed the loss of PTTG3P abolished the miR-132/212-3p-knockdown-induced increase of luciferase activity of FoxM1-WT 3′-UTR, while the mutated 3′-UTR of FoxM1 did not show a response to miR-132/212-3p (Fig. 6d). These results were further confirmed at both the mRNA (Fig. 6e) and protein levels (Fig. 6f) of FoxM1. Collectively, these data supported that PTTG3P functions as a molecular sponge for miR-132/212-3p to facilitate expression of FoxM1.

### Transcriptional activation of PTTG3P by FOXM1

**Fig. 7:**
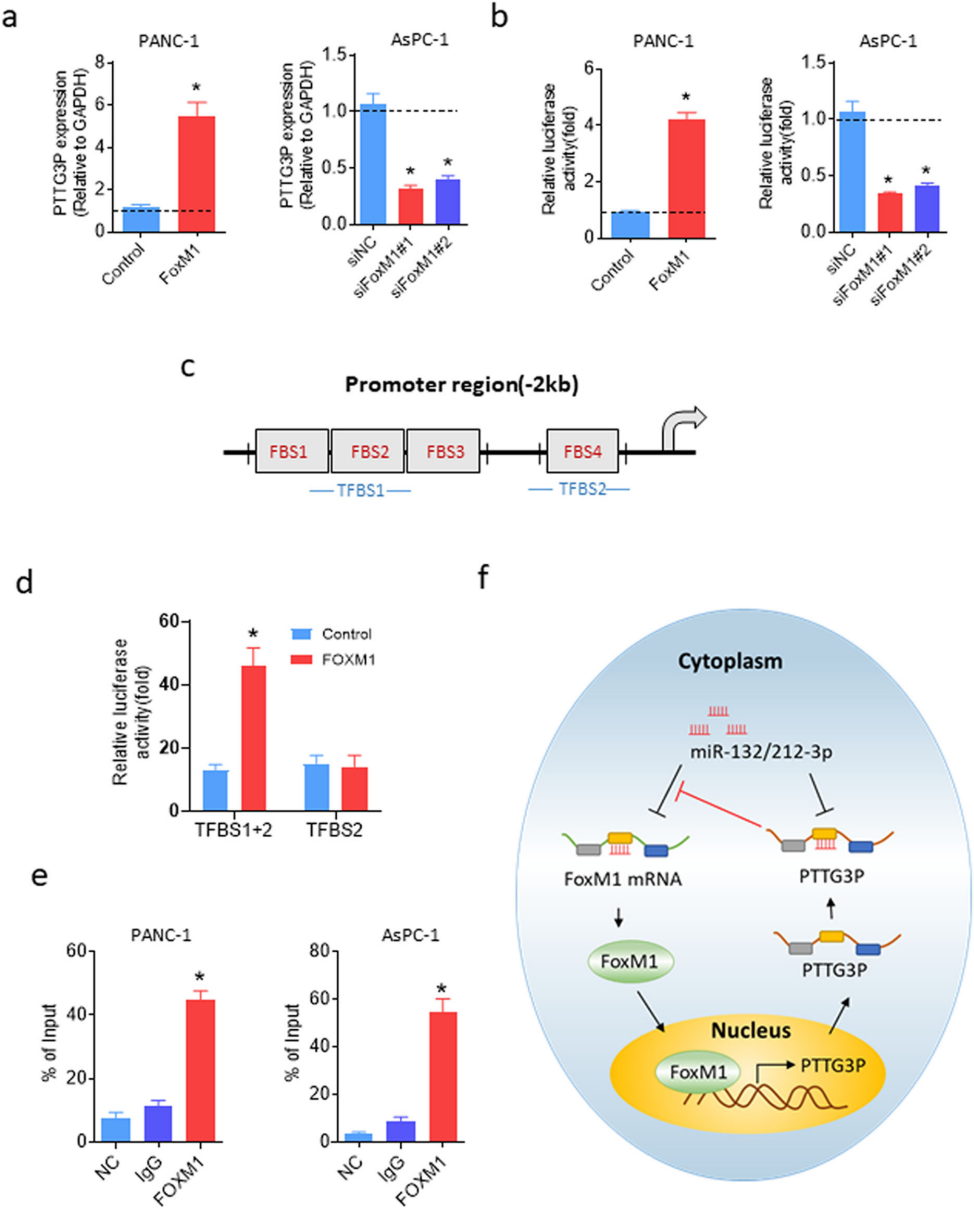
PTTG3P was transcriptionally activated by FoxM1. **a** The qRT-PCR analysis revealed upregulation of PTTG3P in the PANC-1 cells transiently transfected with FoxM1(left) and reduction of PTTG3P in the AsPC-1 cells following the depletion of endogenous FOXM1 by siRNA(#1 and #2)(right). **b** The ability of FoxM1 to bind promoter region of PTTG3P were confirmed in PANC-1 and AsPC-1 cells. **c** A schematic diagram illustrating the PTTG3P promoter luciferase reporter constructs: TFBS1 with FBS1-FBS3 and TFBS2 only with FBS4. TFBS, transcription binding site. FBS, FoxM1 binding site. **d** The luciferase promoter assay in 293T cells demonstrated FOXM1 transcriptionally activated PTTG3P by binding to the TFBS1, instead of TFBS2. **e** The ChIP assay demonstrated that PCR fragments covering TFBS1 were pulled down with the FOXM1 antibody in both PANC-1(left) and AsPC-1 cells (right). **f** Schematic model shows the results of the study. *P < 0.05, Means ± SD was shown. The experiments were performed in triplicate.

We further investigated the underlying mechanism for PTTG3P upregulation in PDAC cells. Intriguingly, bioinformatics analysis of promoter region of PTTG3P predicted multiple potential binding sites for FoxM1 (Supplementary Fig. 4). It showed that ectopic expression of FoxM1 increased PTTG3P, whereas FoxM1 knockdown decreased PTTG3P in PDAC cells (Fig. 7a). As shown in Fig. 7b, increased FOXM1 expression in PANC-1 cells significantly upregulated PTTG3P promoter activity, whereas knockdown of FOXM1 expression in AsPC-1 cells showed obvious inhibitory effect. Three canonical FOXM1-binding sites (FBSs) (5′-CACCC-3′ and 5′-(G/A)(G/A)GG(C/T)G(C/T)-3′) were identified in the PTTG3P promoter region, locating at -1009 (FBS1), -1001 (FBS2), -993 (FBS3) and -192 (FBS4) bp relative to the transcriptional start site of PTTG3P (Fig. 7c). For that first three FBSs (FBS1, 2 and 3) were closely adjacent, we constructed reporters with full-length PTTG3P promoter PTTG3P-1009 (Covering all FBSs) and a deletion mutants (PTTG3P-192, covering FBS4) of it. Reporters with or without FOXM1 expression vectors were transfected into 293 T cells. Luciferase reporter assay results demonstrated that deletion of the region covering FBS1-3 markedly decreased the promoter activity of PTTG3P activated by FOXM1 (Fig. 7d). Chromatin immunoprecipitation (ChIP) assay also confirmed FoxM1 directly bound TFBS1 in both PANC-1 and AsPC-1 cells (Fig. 7e). These data suggested that activation of PTTG3P, at least partially, was resulted from the upregulation of FoxM1 in PDAC cells.

## Discussion

Accumulating evidence supports a crucial role of pseudogenes, a new class of lncRNAs, in both physiological and pathological processes. For instance, HK2P1, a processed pseudogene of HK2 involved in glucose metabolism, has been demonstrated to post-transcriptionally modulates the expression of HK2 and then be actively involved in preeclampsia^16,17^. Pseudogene PTENP1 inhibited hepatocellular carcinoma (HCC) progression *via* inducing PTEN expression, an important tumor suppressor gene^18^. In this study, we demonstrated that a pseudogene, PTTG3P, was positively correlated with tumor aggressiveness and promoted cancer progression *via* a PTTG3P/miR-132/212-3p/FoxM1 feedback loop (Fig. 7e).

PTTG3P was first regarded as a protein coding gene but proved to be a pseudogene that was unable to encode fully functional protein by following automated computational analysis^19^. Dysregulation of PTTG3P has been demonstrated in many gastrointestinal and other human cancers. For example, by using TCGA database, PTTG3P was found to be upregulated in gastric cancer tissues and served as an independent negative prognostic predictor^20^. Huang et al.^11^ observed an elevated expression of PTTG3P in HCC tissues by microarrays and found its level was positively correlated with tumor size, TNM stage and poor survival of HCC patients. Similar results were also reported in cervical carcinoma and breast cancer cohorts^11,21^ However, role of PTTG3P in PDAC has yet been well understood. Here, we discovered, for the first time, the correlation of PTTG3P with clinical characteristics of PDAC patients. It showed that PTTG3P was frequently upregulated in PDAC tissues relative to corresponding non-cancerous controls. Clinical data revealed that elevated PTTG3P closely correlated with larger tumor size, poorer differentiation and reduced overall survival. These findings were also supported by bioinformatics analysis by using several online PDAC datasets.

Bioeffect of PTTG3P in cancers has been explored in previous studies. A genome-scale RNA-mediated interference profiling of cell division in HeLa cells showed that depletion of PTTG3P through specific siRNA results in G0/G1 arrest^22^. It reported that PTTG3P may participate in the chemoresistance to 5-fluorouracil. Treatment of 5-fluorouracil decreased PTTG3P expression in tumor cells, and enhanced-expression of PTTG3P partially attenuated 5-fluorouracil-induced cell apoptosis^8,23^. Moreover, PTTG3P has been demonstrated to promote tumor cell proliferation and invasion in gastric and liver cancers^12,20^ In order to explore the biofunction of PTTG3P in PDAC cells, we performed gain of- and loss of function experiments in PDAC cell lines. Overexpression of PTTG3P markedly increased cell proliferation, migration and invasion in cultured PDAC cells, while knockdown of PTTG3P did the opposite. In vivo evidence using xenograft mouse models confirmed our findings in vitro. These collective results consistently established the notion that high PTTG3P expression is a decisive factor in driving human PDAC development and progression.

Elucidating the molecular basis of PTTG3P-induced protumor effect contribute to the development of rationally designed combination therapies to compensatory signaling pathways. Huang et al.^8^ demonstrated that PTTG3P drive HCC progression *via* activating PI3K/AKT signaling. PTTG1 has been demonstrated to be a key target of PTTG3P in both breast cancer and HCC^8,11^. In a breast cancer cohort, Lou et al.^21^ demonstrated an elevated expression pattern of PTTG3P and discovered that PTTG3P expression correlated negatively with estrogen receptor (ER) and progesterone receptor (PR) status. A recent study reported that PTTG3P upregulated the expression of Cyclin D1 (CCND1) and poly ADP-ribose polymerase 2 (PARP2) by sponging miR-383, acting as a ceRNA. However, current knowledge of PTTG3P in PDAC is quite limited.

It has been well established that FoxM1 is a transcription factor critical to tissue development and cell proliferation^24^. Our previous study identified five FOXM1 isoforms present in pancreatic cancer, among which FOXM1c was predominantly expressed and significantly drive PDAC malignant progression^25^. A more recent study by our group demonstrated that loss of miR-494 induce FoxM1 expression, thus activing nuclear translocation of β-catenin and its downstream pathways^14^. Shih et al.^26^ reported PTTG3P could interact directly with FoxM1 protein to promote its transcriptional activity. In this study, we found PTTG3P activated the expression of FoxM1 in terms of RNA and protein levels. All these findings suggest an important trigger effect of PTTG3P on activation of FoxM1 signaling.

Given that PTTG3P was a predominantly cytoplasmic located lncRNA, we next investigated whether it act as a ceRNA to sequester certain microRNAs targeting FoxM1. Bioinformatics analyses and experimental evidence revealed miR-132/212-3p, which could be sponged by PTTG3P, were critical regulators of FoxM1. The miR-132-3p and miR-212-3p, two microRNAs share close sequences highly conserved among vertebrates. Their expression is necessary for the proper development, maturation and function of cells and new evidence points towards a role of these microRNAs in cancer development and progression. According to previous reports, biofunctions of miR-132-3p and miR-212-3p were diverse in PDAC, showing either tumor promoter or tumor suppressor effects^27–30^. A recent study found overexpression of the miR-132/212-3p cluster reduces development of doxorubicin-induced cardiotoxicity^31^. In this study, we discovered a new suppressive role of miR-132/212-3p by targeting oncogene FoxM1. Given the common target identified in this work, it is interesting for further studies to determine the extent to which the functions of miR-132-3p and miR-212-3p are overlapping.

To our knowledge, mechanism responsible for PTTG3P upregulation has not been reported to date. Our work identified human PTTG3P as a FoxM1-inducible lncRNA and detected the specific binding sites in its promoter region. Recent studies showed lncRNAs, including LINC-ROR, TUG1, Snhg8 and Gm26917, could be transcriptionally activated by FoxM1^32–34^. These studies, together with ours, suggesting that lncRNAs, which are previously unappreciated molecules, may play critical roles in the regulation of FoxM1-induced signaling.

In summary, our study demonstrated that pseudogene PTTG3P was upregulated in PDAC tissues and acted as an oncogene to promote tumor growth and metastasis in vitro and in vivo. We also identified PTTG3P, as a downstream target of FoxM1, competitively binds to miR-132/212-3p to abolish their suppressive effects on FoxM1, thus forming a potent feedback loop to drive PDAC aggressiveness. These results suggested that targeting the PTTG3P/miR-132/212-3p/FOXM1 loop might be a useful strategy for future cancer treatment in PDAC patients.

## Supplementary information

Supplementary Figure 1

Supplementary Figure 2

Supplementary Figure 3

Supplementary Figure 4

STable 1

STable 2

STable 3

STable 4

STable 5
