# Supplementary figures and images for: A novel lncRNA PTTG3P/miR-132/212-3p/FoxM1 feedback loop facilitates tumorigenesis and metastasis of pancreatic cancer
Source: Cell Death Discov. 2020 Nov 30;6:136. doi: 10.1038/s41420-020-00360-5 (PMC7705684; doi:10.1038/s41420-020-00360-5)

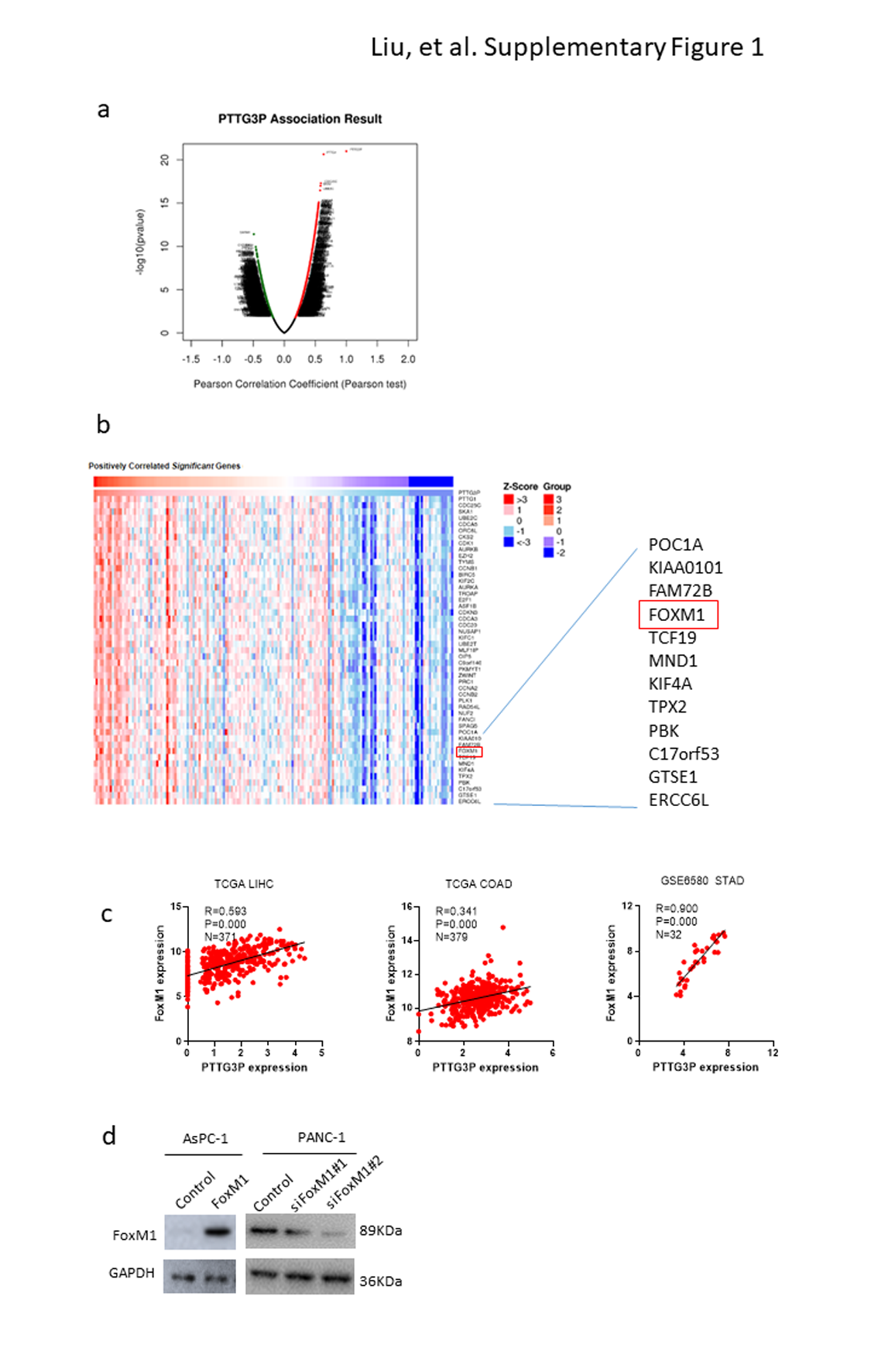

Supplement: Supplementary file 1 — Supplementary Figure 1 [file 41420_2020_360_MOESM1_ESM.tif]

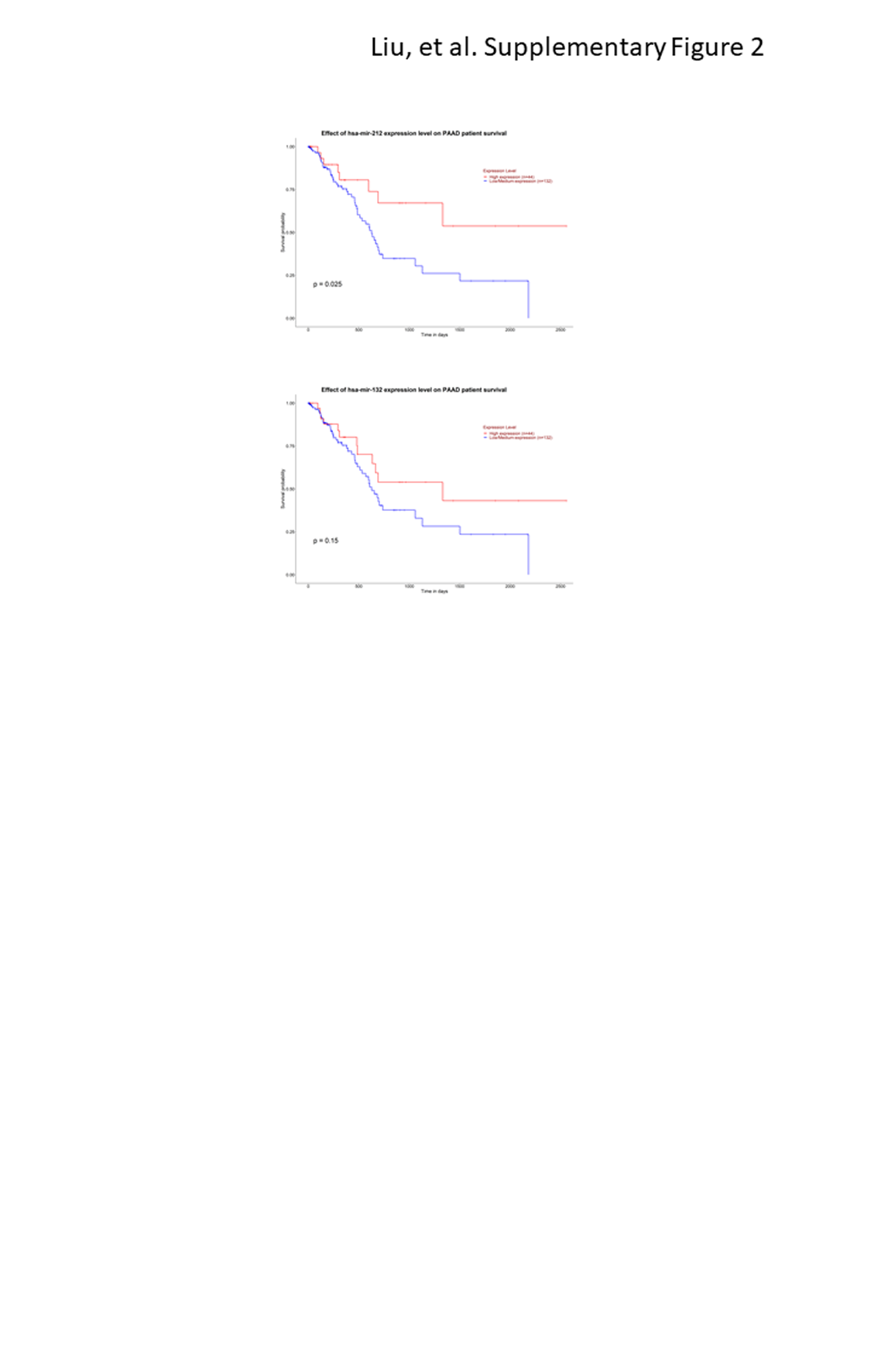

Supplement: Supplementary file 2 — Supplementary Figure 2 [file 41420_2020_360_MOESM2_ESM.tif]

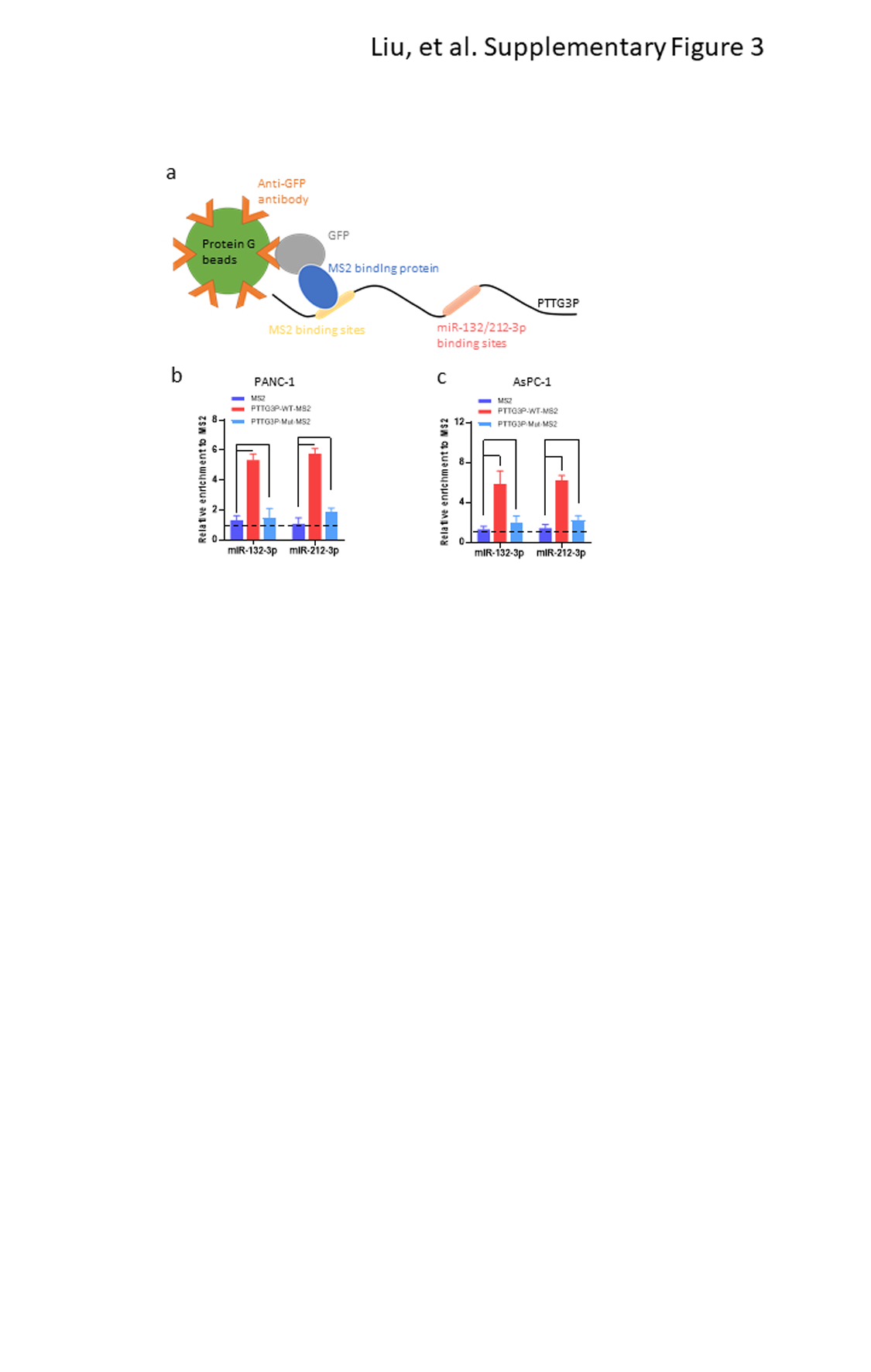

Supplement: Supplementary file 3 — Supplementary Figure 3 [file 41420_2020_360_MOESM3_ESM.tif]

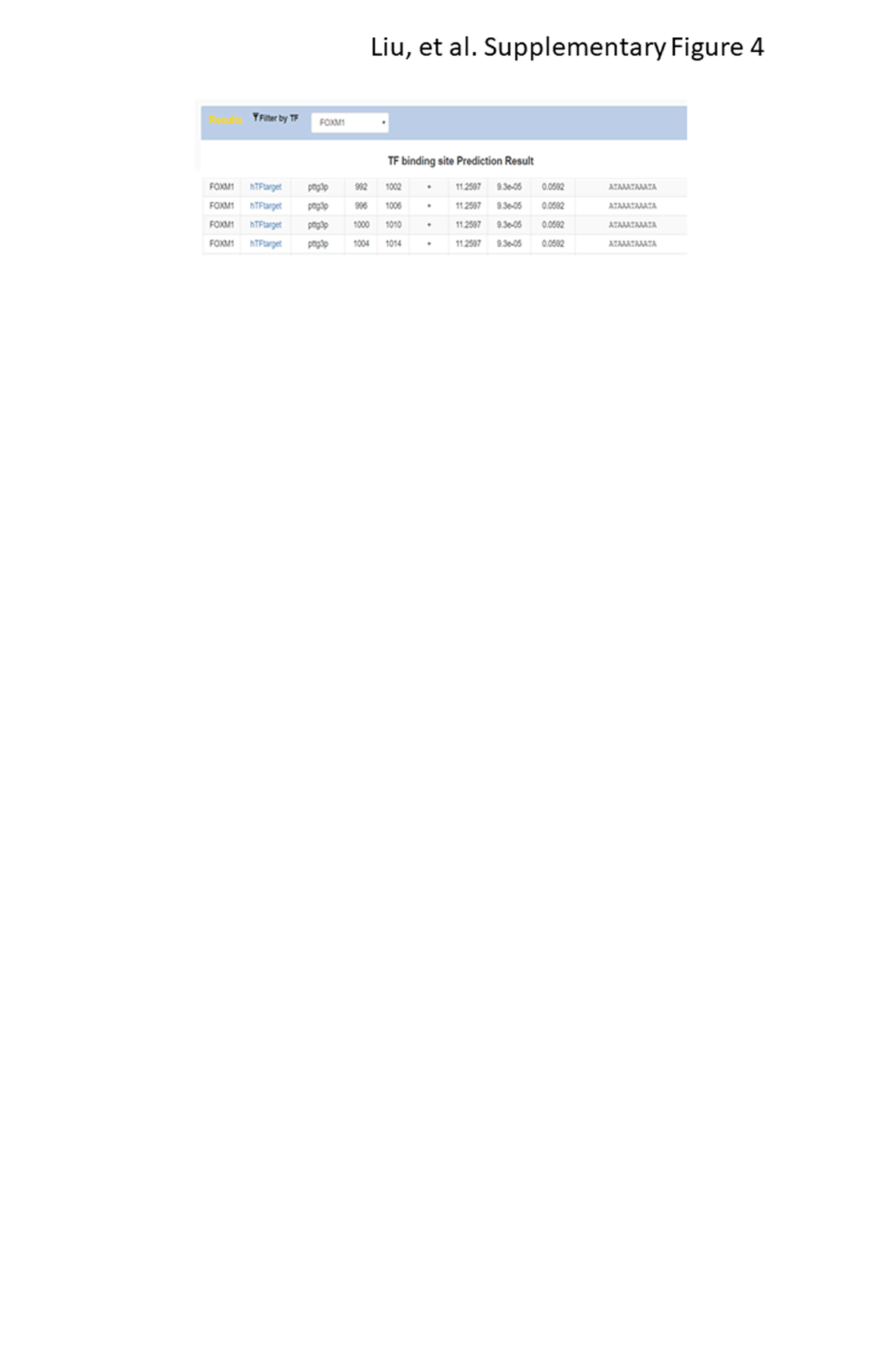

Supplement: Supplementary file 4 — Supplementary Figure 4 [file 41420_2020_360_MOESM4_ESM.tif]
