# Supplementary material for: A novel lncRNA PTTG3P/miR-132/212-3p/FoxM1 feedback loop facilitates tumorigenesis and metastasis of pancreatic cancer
Source: Cell Death Discov. 2020 Nov 30;6:136. doi: 10.1038/s41420-020-00360-5 (PMC7705684; doi:10.1038/s41420-020-00360-5)
Supplement: Supplementary file 5 — STable 1 [file 41420_2020_360_MOESM5_ESM.docx]

**Supplementary Table 1. Clinicopathological characteristics of the PDAC patients in cohort 1 (*n* = 25)**

| **Characteristic** | ***n*** | **%** |
| --- | --- | --- |
| Sex |  |  |
| Male | 16 | 64 |
| Female | 9 | 36 |
| Age, years |  |  |
| ≤60 | 18 | 72 |
| >60 | 7 | 28 |
| Depth of invasion |  |  |
| T1 | 2 | 8 |
| T2 | 16 | 64 |
| T3 | 7 | 28 |
| Differentiation |  |  |
| Well | 2 | 8 |
| Moderate | 15 | 60 |
| Poor | 8 | 32 |
| Tumor size |  |  |
| ≤3 cm | 10 | 40 |
| >3 cm | 15 | 60 |
| N stage |  |  |
| N0 | 12 | 48 |
| N1 | 13 | 52 |
| Distant metastasis |  |  |
| No | 23 | 92 |
| Yes | 2 | 8 |
