# Supplementary material for: A novel lncRNA PTTG3P/miR-132/212-3p/FoxM1 feedback loop facilitates tumorigenesis and metastasis of pancreatic cancer
Source: Cell Death Discov. 2020 Nov 30;6:136. doi: 10.1038/s41420-020-00360-5 (PMC7705684; doi:10.1038/s41420-020-00360-5)
Supplement: Supplementary file 6 — STable 2 [file 41420_2020_360_MOESM6_ESM.docx]

**Supplementary Table 2. Correlation of clinicopathological variables with PTTG3P expression in PDAC patients from cohort 2 (n=60).**

|  | |  |  | PTTG3P  expression | | P value |
| --- | --- | --- | --- | --- | --- | --- |
| Clinicopathological  Feature | No. of  Patients | | Percent  (%) | High  (n=30) | Low  (n=30) |  |
| Gender | |  |  |  |  | 0.288 |
| Male | | 37 | 62 | 21 | 16 |  |
| Female | | 23 | 38 | 9 | 14 |  |
| Age | |  |  |  |  | 0.779 |
| ≤60 | | 18 | 30 | 10 | 8 |  |
| >60 | | 42 | 70 | 20 | 22 |  |
| Tumor size | |  |  |  |  | 0.020* |
| ≤3 cm | | 17 | 28 | 4 | 13 |  |
| >3 cm | | 43 | 72 | 26 | 17 |  |
| T category | |  |  |  |  | 0.577 |
| T1 | | 4 | 7 | 1 | 3 |  |
| T2 | | 43 | 72 | 22 | 21 |  |
| T3 | | 13 | 21 | 7 | 6 |  |
| N stage | |  |  |  |  | 0.592 |
| N0 | | 38 | 63 | 18 | 20 |  |
| N1 | | 22 | 37 | 12 | 10 |  |
| Distant metastasis | |  |  |  |  | 1.000 |
| No | | 58 | 97 | 28 | 30 |  |
| Yes | | 2 | 3 | 2 | 0 |  |
| Differentiation | |  |  |  |  | 0.006* |
| Well | | 10 | 17 | 3 | 7 |  |
| Moderate | | 33 | 55 | 13 | 20 |  |
| Poor | | 17 | 28 | 14 | 3 |  |

Differences among variable were assessed by chi-square test.*, the values had statistical significant differences.
