# Supplementary material for: A novel lncRNA PTTG3P/miR-132/212-3p/FoxM1 feedback loop facilitates tumorigenesis and metastasis of pancreatic cancer
Source: Cell Death Discov. 2020 Nov 30;6:136. doi: 10.1038/s41420-020-00360-5 (PMC7705684; doi:10.1038/s41420-020-00360-5)
Supplement: Supplementary file 7 — STable 3 [file 41420_2020_360_MOESM7_ESM.docx]

**Supplementary Table 3. Oligonucleotide sequences**

| **Gene name** | **Forward primer (5’-3’)** | **Reverse primer (5’-3’)** |
| --- | --- | --- |
| Primers for real-time quantitative PCR | | |
| PTTG3P | AATCTGGTTGAGAGCGGCAA | CAGCCCATCCTTTGTAGCCA |
| FoxM1 | ACGTCCCCAAGCCAGGCTC | CTACTGTAGCTCAGGAATAA |
| GAPDH | TCACCACCAT GGAGAAGGC | GCTAAGCAGTTGGTGGTGCA |
| U6 | GAATTCCCCAGTGGAAAGACGC | GGTGTTTCGTCCTTTCCACAAGATATATAAAGGG |
| Primers for PTTG3P promoter constructs | | |
| TFBS1+2 (-1047~-1) | ATTTCAGCCTGGGCGACAGAGCAAG | CTGTTTCAGCTGGGCCCCCCTTCAC |
| TFBS2 (-222~-1) | GATTTAAGTATATATCTTTTTTTCT | CTGTTTCAGCTGGGCCCCCCTTCAC |
| Primers for ChIP assay | | |
| TFBS1(-1045~-876) | TTCAGCCTGGGCGACAGAGCAAG | CCCACAATTATAGTTAGAAGTTTCA |
| TFBS2(-303~-139) | CATTATTATCCCATTTTATGTCCTT | ATTTCTTAGTGCTTTAGAGTTTGTG |
